# Diabetic Eye Screening Attendance Among Minority Ethnic and Other Inequality-Affected Populations in the United Kingdom: A Systematic Review of Associated Factors, Barriers, Facilitators and Interventions

**DOI:** 10.3390/vision10040066

**Published:** 2026-09-04

**Authors:** Hammaad Khalid, Farah Andaleeb

**Affiliations:** Medicine, Leeds Teaching Hospitals NHS Trust, Leeds LS1 3EX, UK

**Keywords:** diabetic eye screening, diabetic retinopathy screening, ethnicity, minority ethnic communities, health inequalities, deprivation, attendance, non-attendance, National Health Service

## Abstract

Diabetic retinopathy is a leading cause of preventable sight loss. The United Kingdom achieves high overall uptake through organised diabetic eye screening programmes, but aggregate performance may conceal inequalities. This systematic review synthesised United Kingdom evidence on attendance-related associated factors, barriers, facilitators and interventions, with particular emphasis on minority ethnic communities and related inequalities. MEDLINE and Embase via Ovid, Web of Science, Scopus, the Cochrane Central Register of Controlled Trials, Google Scholar, citation searching and targeted United Kingdom grey-literature sources were searched and updated to 18 August 2026. Fourteen primary studies met the inclusion criteria. Younger age and socioeconomic deprivation showed the most consistent associations with lower or delayed attendance across multiple large routine-data analyses. Ethnicity-related associations varied by group and setting, and several datasets were limited by incomplete ethnicity recording. Qualitative, mixed-methods and survey evidence identified communication difficulties, competing work or education, appointment inflexibility, transport, inaccurate contact details and fragmentation with diabetes care as plausible barriers. One cluster-randomised trial in practices serving South Asian communities increased mean practice-level attendance from 74% to 89% (adjusted difference 12 percentage points, 95% confidence interval 7–17). Overall confidence was strongest for age- and deprivation-related associations and lower for specific mechanisms and interventions. Equity improvement should combine robust demographic monitoring with locally demonstrated barriers and the evaluation of intervention reach and sustained attendance.

## 1. Introduction

Diabetes mellitus (DM) is a major and growing public-health challenge in the United Kingdom (UK), affecting approximately six million people. Its distribution and complications are shaped by age, ethnicity and socioeconomic circumstances, making equitable access to preventive care an important component of diabetes management [1].

Diabetic retinopathy (DR) is a potentially sight-threatening microvascular complication of DM. Disease may progress before visual symptoms develop, so organised retinal screening is intended to identify referable DR and maculopathy early enough for surveillance or treatment [2].

The UK delivers diabetic eye screening through four nation-specific programmes. In England, the National Health Service (NHS) programme systematically identifies eligible people aged 12 years or older and invites them through a call-and-recall pathway supported by national standards, quality assurance and referral arrangements [3]. Similar organised programmes operate in Scotland, Wales and Northern Ireland, although service configuration and recall arrangements differ.

Overall programme performance is high. England achieved nationwide coverage by 2008, and 2.23 million of 2.70 million invitees were screened in 2017–2018 (82.7%). Screening has contributed, alongside improved DM management and treatment, to reductions in sight loss attributed to DR [4]. For context, a 2024 meta-analysis estimated pooled screening adherence of 66.9% in high-income countries and 39.3% in low- and middle-income countries [5]. These estimates are not directly comparable because programmes, denominators and attendance definitions differ, but they show that high aggregate uptake can coexist with important variation in participation.

Equity is therefore distinct from overall uptake. Black African, African Caribbean and South Asian populations experience a higher burden of type 2 diabetes mellitus (T2DM) and may develop disease at younger ages [6], but screening attendance does not follow a uniform ethnic gradient. Earlier systematic reviews identified patient-, provider- and service-level barriers to DR screening, largely from international evidence [7,8]. UK health-equity guidance also emphasises monitoring participation by demographic and socioeconomic characteristics and investigating locally observed gaps [9,10].

Ethnicity may intersect with language, deprivation, age, migration-related circumstances and service design, while incomplete demographic recording can obscure disparities. A UK-specific synthesis is therefore needed to distinguish observed attendance associations from plausible mechanisms and to identify where intervention evidence is available.

This systematic review aimed to synthesise UK primary research on factors associated with diabetic eye screening attendance, reported barriers and facilitators, and interventions among minority ethnic and other inequality-affected populations. The review focused on evidence relevant to equitable access and sustained participation rather than on disease prevalence alone.

## 2. Methods

### 2.1. Review Design

This review examined attendance-related associated factors, barriers, facilitators and interventions in UK diabetic eye screening, with particular emphasis on minority ethnic communities while retaining closely related inequalities that may intersect with ethnicity. The review question was structured using a Population–Interest–Context (PICo) framework: people eligible for diabetic eye screening; factors influencing attendance and responses intended to improve it; and UK diabetic eye screening services (Table 1). An adapted Population–Intervention/Interest–Comparator–Outcome (PICO) eligibility framework is provided in Table A2.

Given variation in study design, populations, associated factors and outcomes, findings were synthesised narratively rather than pooled. The review was conducted and reported in accordance with the Preferred Reporting Items for Systematic Reviews and Meta-Analyses (PRISMA) 2020 guidance [11]. The protocol was not prospectively registered because registration was overlooked and review activities had already commenced when the omission was recognised. This reduces external assurance that all methods were prespecified; the updated search strategy, eligibility criteria, study-level exclusions and design-specific appraisals are therefore reported in full.

### 2.2. Eligibility Criteria

The review was ethnicity-led but not ethnicity-exclusive. UK primary studies were eligible if they reported diabetic eye or DR screening attendance, uptake, non-attendance or access and treated at least one equity-related dimension as a substantive study focus or prespecified analysis. Eligible dimensions were ethnicity or minority ethnic status, language or cultural barriers, socioeconomic deprivation, younger age or life stage, and access-related disadvantage. A demographic variable recorded only incidentally, without a substantive attendance analysis, was insufficient on its own for inclusion.

Primary qualitative, quantitative, mixed-methods, observational and interventional studies were eligible. Studies were excluded if they were conducted outside the UK; did not examine an eligible screening-attendance outcome, associated factor, barrier, facilitator or intervention; focused solely on disease prevalence, diagnostic accuracy, grading, research participation, general diabetes care or post-screening ophthalmology attendance; or lacked a substantive ethnicity or inequality-related focus. Systematic reviews and other secondary evidence were excluded from the primary synthesis but could inform contextual discussion. English-language reports published from 2006 to 18 August 2026 were eligible. Summarised in Table 2.

### 2.3. Search Strategy

The original searches were conducted on 7 June 2026 and were broadened and rerun on 18 August 2026 in MEDLINE and Embase via Ovid. The updated strategy combined controlled vocabulary—Medical Subject Headings in MEDLINE and Emtree in Embase—with free-text terms for diabetic eye or retinopathy screening; attendance, access, barriers, facilitators and equity-related associated factors; and the UK setting. Database-specific adjacency, truncation and field operators were applied. Searches were limited to English-language records published from 2006 to 18 August 2026.

The expanded strategy was also rerun in Web of Science Core Collection, Scopus and the Cochrane Central Register of Controlled Trials (CENTRAL). Six targeted Google Scholar searches covered ethnicity, minority ethnic communities, qualitative barriers, South Asian populations, younger age and socioeconomic deprivation; the first five result pages were screened for each query and 15 potentially relevant records were exported. Backward and forward citation searching of included studies and earlier systematic reviews identified 12 additional records. Full search strings are reported in Appendix A. No eligible UK primary study published in 2025 or 2026 was identified.

Targeted grey-literature searches of GOV.UK and NHS sources identified policy, programme and patient-information documents. No dedicated conference proceedings, thesis or broad grey-literature database was searched. Conference abstracts indexed in the bibliographic databases were not excluded by publication-type filters and were screened, but inclusion required sufficient primary methods and results to establish eligibility and extract findings. Contextual official documents were retained solely to describe current screening systems and interpret service implications; they were not counted as included primary studies or entered into the formal synthesis. Complete database strategies are provided in Table A1; the adapted eligibility framework is in Table A2, and the information sources and record counts are in Table 3.

### 2.4. Study Selection

All identified records were collated and deduplicated before screening. Both reviewers independently screened titles and abstracts against the eligibility criteria and independently assessed retrieved full texts. Disagreements at either stage were resolved through discussion. Reasons for full-text exclusion were recorded, and the process was documented using a PRISMA 2020 flow diagram.

### 2.5. Data Extraction

Data were extracted by H.K. using a standardised form and checked against the source reports by F.A. Extracted fields included author, publication year, design, setting, population and sample size, equity dimension, data-collection methods, attendance and disease outcomes, associated factors, barriers, facilitators, intervention details, effect estimates and study limitations. Discrepancies were resolved through discussion. Attendance outcomes, disease outcomes and professional perceptions were retained as distinct evidence types.

### 2.6. Quality Appraisal and Risk-of-Bias Assessment

Two reviewers independently completed design-specific quality appraisal and risk-of-bias assessment, resolving disagreements through discussion. Joanna Briggs Institute (JBI) checklists were used for qualitative studies, analytical cross-sectional studies and the prevalence-style professional survey [12]; the professional survey checklist was adapted to assess the prevalence of reported beliefs. The cluster-randomised trial was assessed with the Cochrane Risk-of-Bias 2 (RoB 2) tool for cluster-randomised trials [13], and retrospective cohort studies with the Newcastle–Ottawa Scale (NOS) [14]. Mixed-methods studies were appraised according to the components contributing to the synthesis. Standard tool-specific judgements were retained, alongside a cautious narrative overview. No study was excluded solely because of appraisal findings; instead, methodological limitations informed the weight and confidence assigned to each contribution.

### 2.7. Data Synthesis

Due to variation in study design, populations, settings, exposure definitions and outcomes, a structured narrative synthesis was undertaken. Evidence was first separated into attendance or uptake outcomes, disease outcomes, reported barriers or facilitators, professional perceptions and evaluated interventions. Findings were then grouped by recurring equity and service themes. Potentially overlapping cohorts were identified and described separately rather than treated as independent replication.

The synthesis considered socioeconomic deprivation, age and life stage, ethnicity and language, communication and understanding, appointment access, transport, service organisation, reminder systems, proactive contact and integration with routine diabetes care. Adjusted estimates were prioritised where available. Disease outcomes were used to interpret whether attendance corresponded to clinical risk, not as substitutes for attendance outcomes. Findings from the professional survey were reported as perceptions rather than evidence of intervention effectiveness.

Meta-analysis was not performed because the included studies were heterogeneous in design, population characteristics, exposure definitions and outcome measures, and did not report sufficiently comparable data for pooled quantitative analysis.

### 2.8. Certainty of Evidence

A formal outcome-specific Grading of Recommendations Assessment, Development and Evaluation (GRADE) assessment was not undertaken because this review did not aim to estimate a pooled intervention effect and included heterogeneous qualitative, observational, mixed-methods and interventional evidence. The included studies reported varied outcomes relating to screening attendance, uptake, access, barriers and facilitators, which limited the feasibility of applying a single outcome-specific certainty framework.

Instead, confidence in each principal finding was considered narratively using methodological limitations, directness to the review question, consistency across studies, precision where estimable and the independence of datasets. Confidence was reduced where evidence relied on a single study, professional perceptions, area-level proxies, small samples, incomplete demographic data or partly overlapping cohorts. Overall conclusions remain cautious because only 14 studies were included and ethnicity-specific and intervention evidence was limited.

## 3. Results

### 3.1. Study Selection

The updated searches identified 1012 records: 1000 from bibliographic databases and Google Scholar and 12 through backward or forward citation searching. After 429 duplicates were removed, 583 records underwent title and abstract screening and 535 were excluded.

Forty-eight full-text reports were retrieved and assessed. Thirty-four were excluded: five lacked a substantive ethnicity or inequality focus, 16 lacked an eligible attendance-related associated factor, barrier, facilitator or intervention, four were secondary evidence and nine involved the wrong population or location. Fourteen primary studies were included in the narrative synthesis.

**Figure 1:**
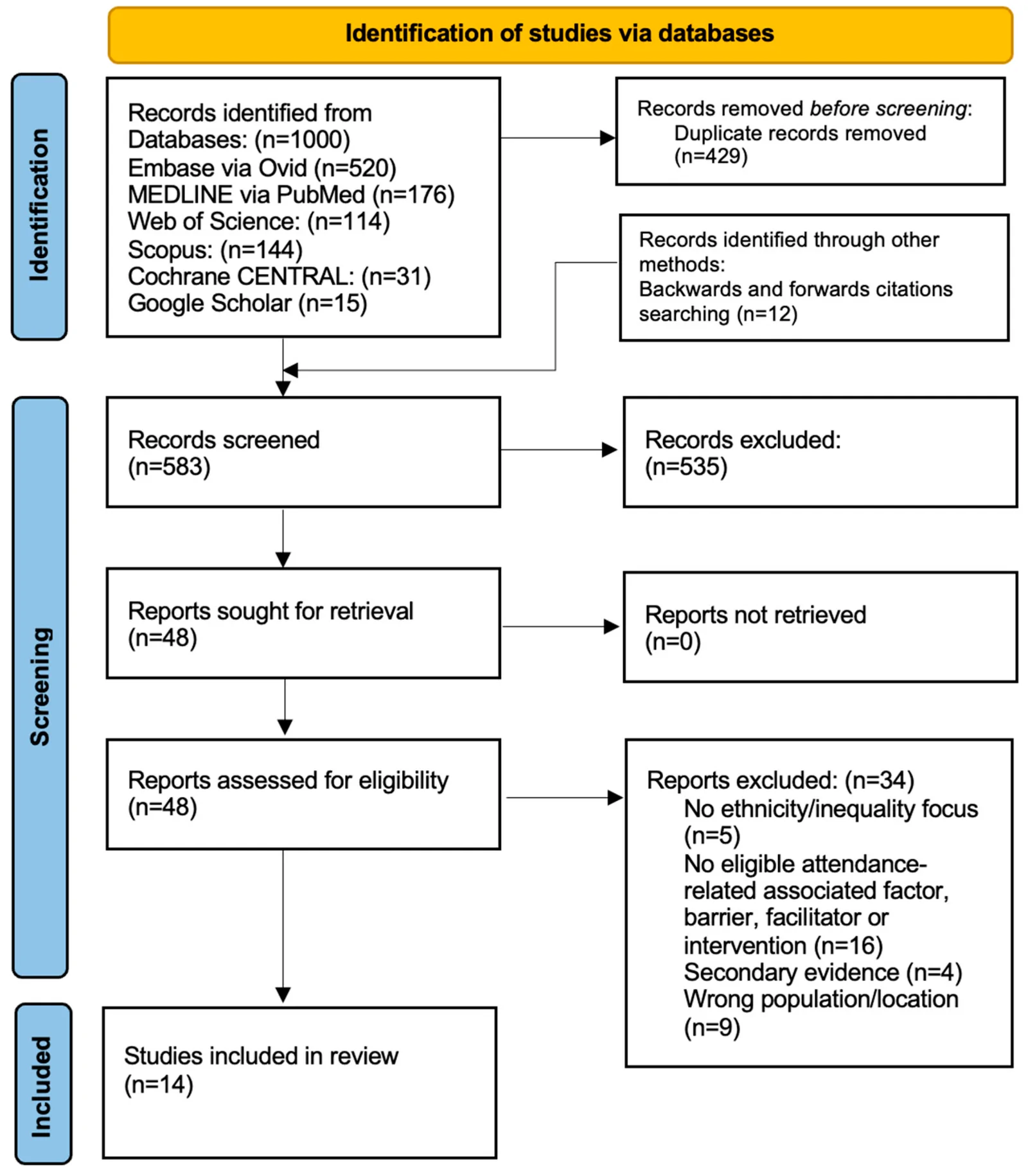
Preferred reporting items for Systematic Reviews and Meta-Analyses 2020 flow diagram illustrating the study selection process [11].

Ten official UK programme documents were retained for background and policy context only and were not included in the primary-study synthesis. The selection process is shown in Figure 1 and summarised in Table 4.

All 34 full-text exclusions and study-specific reasons are reported in Supplementary Table S2.

### 3.2. Study Characteristics

Fourteen primary studies met the inclusion criteria: six cross-sectional or health-equity audits, two qualitative studies, two mixed-methods studies, three retrospective cohort studies and one cluster-randomised proof-of-concept trial, published between 2006 and 2021. Eleven studies were England-specific, one was conducted in Northern Ireland, one used national Welsh patient-level data and one surveyed screening professionals across the UK. Sample sizes ranged from 25 questionnaire respondents to 291,296 eligible participants. The studies addressed ethnicity, deprivation, age and life stage, communication and access, persistent non-attendance, and service organisation and one evaluated intervention. Full characteristics are presented in Table 5.

### 3.3. Quality Appraisal and Risk of Bias

Methodological robustness varied by design and outcome. The retrospective cohorts by Lawrenson et al. and Thomas et al. were judged comparatively lower concern on the NOS (8/9 each), while Olvera–Barrios et al. scored 7/9 and retained some concerns [18,19,28]. Among analytical cross-sectional studies, Moreton et al. and Gulliford et al. provided relatively strong adjusted routine-data analyses [22,24]. Scanlon et al., Millett and Dodhia, Ahmad and Neilson, and Orton et al. retained concerns related to area-level deprivation measures, incomplete demographic data, descriptive or mixed-methods components, or non-response [15,25,26,27]. These studies support associations but not causal explanations.

Qualitative and survey evidence provided explanatory and service-level insight but carried important limitations. Lindenmeyer et al. sampled nine practices and reported limited researcher reflexivity [16]. Strutton et al. obtained reasons for 146 of 258 eligible never-attenders, with responses recorded as staff notes rather than verbatim transcripts [21]. Cushley et al. included only 25 questionnaire respondents and under-represented persistent non-attenders [20]. Prothero et al. used a theory-informed national survey, but the response rate was 24.1% and reported strategy effectiveness reflected professional perception rather than measured attendance effects [23]. Orton et al. added patient-reported information, but the postal questionnaire response rate was 43% and only 32 non-attenders were interviewed [27].

**Figure 2:**
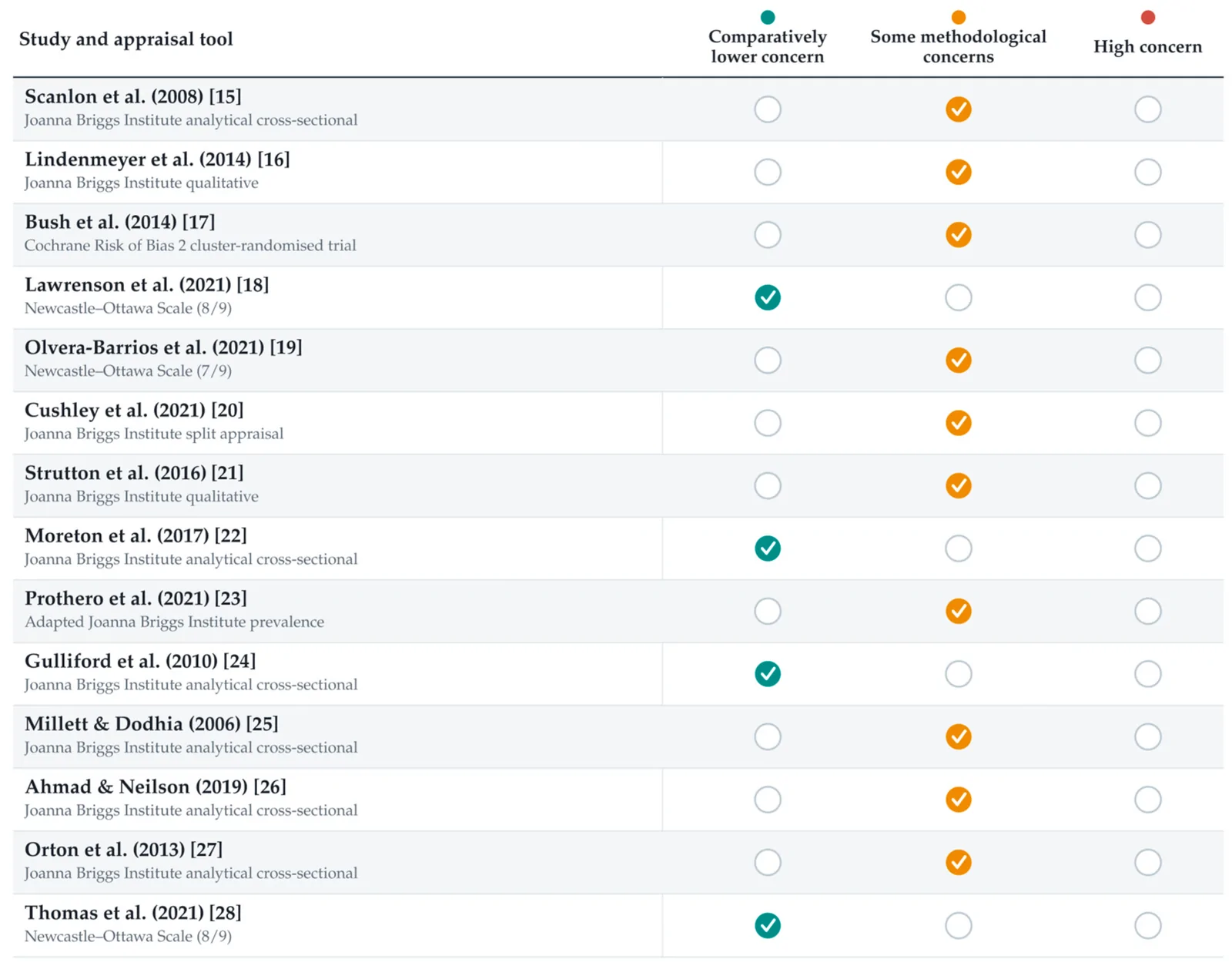
Overall methodological appraisal of the 14 included studies. Each study carries a single tick in one of three categories: green, few or no unmet items on the relevant appraisal tool; amber, one or more unmet or unclear items, so findings were treated as supportive rather than definitive; red, high concern, a category no study met. Judgements were derived from the design-specific tool named beneath each study and are shown for visual overview only; categories are not directly interchangeable across JBI, NOS and RoB 2 assessments. Item-level assessments appear in Table A3, Table A4, Table A5, Table A6 and Table A7.

The Bush et al. cluster trial was judged to raise some concerns because only ten practice clusters were included, baseline imbalance was possible and intervention reach depended on successful contact [17]. No study was excluded on methodological grounds. Appraisal findings instead determined the confidence placed in each contribution and are summarised in Table 6 and Figure 2; full tool-specific assessments appear in Table A3, Table A4, Table A5, Table A6 and Table A7, with the contextual grey literature listed in Table A8.

### 3.4. Narrative Synthesis of Attendance Inequalities, Barriers and Facilitators

#### 3.4.1. Socioeconomic Deprivation and Attendance

Socioeconomic deprivation was associated with lower screening attendance across most studies that examined it. Scanlon et al. [15] reported a decline in uptake from 76.7% in the least deprived quintile to 67.4% in the most deprived quintile. Lawrenson et al. [18] and Olvera–Barrios et al. [19] also identified deprivation as independently associated with non-attendance in large urban English cohorts. Moreton et al. [22] found lower attendance with greater practice-level deprivation, while Gulliford et al. [24] similarly reported greater deprivation to be associated with non-attendance in South London.

Millett and Dodhia [25] reported an OR for attendance of 0.58 (95% CI 0.36–0.95) for participants in the most versus least deprived quintile. Orton et al. [27] similarly found greater deprivation independently associated with non-uptake (OR 1.19, 95% CI 1.10–1.29). Ahmad and Neilson [26] observed lower uptake in more deprived groups across all six programmes examined, and Thomas et al. [28] found greater deprivation independently associated with repeat non-attendance in the national Welsh cohort, in which repeat non-attendance occurred in 18% of participants with T1DM and 8% of those with T2DM. The smaller Northern Ireland youth study [20] was the exception, reporting no significant deprivation association.

#### 3.4.2. Ethnicity and Screening Attendance

Ethnicity-related attendance patterns varied between groups and settings. Olvera–Barrios et al. [19] reported an overall attendance rate of 83.4% in North East London; after adjustment, South Asian, Chinese and other Asian participants had higher odds of attendance than White British participants, while attendance among Black participants was similar. Lawrenson et al. [18] likewise found higher adjusted attendance among Asian or Asian British participants, no significant difference among Black or Black British participants, and lower attendance among mixed-ethnicity participants across three urban English screening programmes.

Gulliford et al. [24] directly modelled self-reported ethnicity alongside deprivation, age and clinical factors in South London, whereas Millett and Dodhia [25] found no statistically significant association between the region of birth and screening attendance. Interpretation was limited in some studies by incomplete ethnicity recording; Ahmad and Neilson [26], for example, could not analyse ethnicity reliably for this reason.

Clinical risk did not necessarily follow the same pattern as attendance. Lawrenson et al. [18] reported greater odds of referable DR among Asian or Asian British and Black or Black British attendees, while Millett and Dodhia [25] found higher DR prevalence among several overseas-born groups despite no significant attendance association with region of birth. Disease outcomes were therefore retained as separate contextual outcomes rather than being used as proxies for screening attendance.

#### 3.4.3. Younger Age and Life-Stage Barriers

Younger age was the most consistently reported attendance-related factor. Lawrenson et al. [18] and Olvera–Barrios et al. [19] identified younger age as independently associated with non-attendance in large English cohorts. Moreton et al. [22] reported overall attendance of 82.4%, falling to 66.6% among those aged 12–39 years, while Gulliford et al. [24] and Millett and Dodhia [25] also found lower attendance among younger participants.

Ahmad and Neilson [26] found uptake below 70% among people aged 19–44 years in all six screening programmes examined. Orton et al. [27] reported younger age to be independently associated with non-uptake, and Thomas et al. [28] found younger age independently associated with repeat non-attendance in both T1DM and T2DM cohorts. Cushley et al. [20], focusing specifically on people aged 12–26 years in Northern Ireland, also found age among the factors associated with screening engagement.

#### 3.4.4. Access, Appointment Timing and Practical Barriers

Practical barriers to attendance were reported across qualitative, mixed-methods and survey studies. Lindenmeyer et al. [16] identified transport difficulties, geographical access and inflexible appointment systems as barriers to uptake. Strutton et al. [21] reported competing commitments, anxiety and practical problems associated with inaccurate or poorly shared patient information among persistent never-attenders.

Among younger participants, Cushley et al. [20] identified inconvenient appointment times, work or school commitments and access difficulties. Prothero et al. [23] similarly found that screening professionals perceived accessibility, scheduling and rescheduling difficulties as barriers for younger adults. Orton et al. [27] added patient-reported evidence of access-related difficulties within a mixed-methods health-equity audit.

Thomas et al. [28] found that more frequent residential moves were independently associated with repeat non-attendance in both T1DM and T2DM cohorts. This was the principal quantitative evidence linking residential instability with repeated failure to attend screening.

#### 3.4.5. Communication and Understanding

Communication and understanding were recurrent barriers across qualitative, mixed-methods and survey evidence. Lindenmeyer et al. [16] identified English-only materials, unmet language needs and communication failures between general practices and screening services. Strutton et al. [21] reported misinformation together with inaccurate or unshared address, as well as clinical and practical information among persistent never-attenders.

Cushley et al. [20] found that some younger participants wanted clearer explanations of how diabetes affects the eyes and reported poor communication as a barrier. In the health-equity audit by Orton et al. [27], 28% of questionnaire respondents did not recall discussion of the importance of screening with their primary-care team and 11% did not understand the term DR. Prothero et al. [23] additionally found that screening professionals perceived poor communication between providers as a barrier to participation.

No included study used a validated health-literacy measure or quantified the independent effect of communication or language on attendance.

#### 3.4.6. Service Integration, Reminders and Proactive Contact

Service organisation and proactive contact were reported as important influences on attendance across several study designs. Lindenmeyer et al. [16] identified patient contact, integration with diabetes care and processes for newly diagnosed or persistently non-attending patients as practice-level factors affecting uptake. Strutton et al. [21] reported failures in sharing address, clinical and practical information, while Prothero et al. [23] found that screening professionals perceived limited integration with wider diabetes care and poor inter-provider communication as barriers.

Moreton et al. [22] found residual variation in attendance between general practices after adjustment for measured factors, indicating that attendance differed between service settings even after patient and practice characteristics were considered. Thomas et al. [28] found that attendance at the first screening invitation was strongly associated with lower subsequent repeat non-attendance.

Bush et al. [17] provided the only comparative intervention evidence. Mean practice-level attendance was 89% in practices receiving multilingual link-worker support compared to 74% in control practices, with an adjusted difference of 12 percentage points (95% CI 7–17). The intervention combined reminders, encouragement and language-concordant telephone contact, so effects of the individual components could not be separated.

Table 7 summarises the barriers and facilitators that have been identified from the studies.

## 4. Discussion

### 4.1. Principal Interpretation

Across the 14 included studies, younger age and socioeconomic deprivation showed the most consistent associations with lower, delayed or repeated non-attendance [15,18,19,20,22,24,25,26,27,28], whereas ethnicity-related associations varied by group and setting [18,19,24,25]. The pattern across multiple independent routine-data analyses strengthens confidence that age and deprivation identify populations at greater risk of unequal participation, but the largely observational evidence does not establish why these differences occur.

Qualitative, mixed-methods and survey evidence suggests that communication, competing commitments, appointment access, inaccurate contact information and weak integration with diabetes care may contribute to these inequalities [16,20,21,23,27]. The Coventry cluster trial [17] provided the only direct intervention evidence, with improved attendance after multilingual link-worker support, although its multicomponent design prevents attribution to any single element. Overall, the evidence is more consistent with an accumulation of social and service-level barriers than with a single demographic cause.

### 4.2. Ethnicity and Screening Attendance

Ethnicity should be interpreted cautiously. Large adjusted cohorts and South London analyses showed different attendance patterns between ethnic groups rather than a uniform minority ethnic disadvantage [18,19,24,25], while incomplete ethnicity recording limited interpretation in some datasets [26]. These findings argue against using broad minority ethnic categories as a proxy for non-attendance; more granular information on language, deprivation, age and local service access is needed to understand observed differences.

Attendance also does not equate to clinical need. Higher referable DR or DR prevalence was reported in some Asian, Black or overseas-born groups despite similar or higher attendance [18,25]. Qualitative studies also identified language, misinformation and information-sharing problems [16,21], but did not isolate ethnicity as an independent mechanism. Equity monitoring should therefore combine attendance with disease burden and pathway outcomes rather than interpret ethnicity alone as causal.

### 4.3. Socioeconomic Deprivation and Screening Attendance

The consistency of the deprivation gradient across multiple independent settings—including large English routine-data analyses and the national Welsh cohort—supports deprivation as an important marker of screening inequality [15,18,19,22,24,25,26,27,28]. The smaller Northern Ireland youth study was the exception and found no significant association [20].

Most studies measured deprivation at area or practice level rather than at the individual level, so the operative mechanisms remain uncertain. The observed gradient may reflect employment constraints, transport, housing instability, digital exclusion, competing responsibilities or other access barriers rather than deprivation itself. Deprivation is therefore best treated as a marker for the local investigation of pathway friction rather than a causal explanation or proxy for lower motivation.

### 4.4. Younger Age and Screening Attendance

The consistency of the younger-age association across English cohorts and regional programme analyses [18,19,22,24,25,26,27], the Northern Ireland youth study [20], and the Welsh repeat non-attendance cohort [28] suggests that it is not confined to a single screening system or diabetes type. Age itself is not a modifiable mechanism, so the more useful question is why younger people find attendance difficult. Work and education commitments, inconvenient appointment timing, anxiety, communication problems, weak service coordination and residential mobility were reported or perceived across the evidence base [20,21,23,27,28]. Younger age may therefore capture a cluster of life-stage and organisational pressures rather than a single behavioural tendency.

These life-stage pressures provide a rationale for age-tailored access strategies, but their effect on sustained attendance in younger adults requires direct evaluation.

### 4.5. Communication and Understanding

Communication difficulties recurred across qualitative, mixed-methods and survey evidence, including unmet language needs, misinformation, unclear explanations, inaccurate patient information and poor communication between providers [16,20,21,23,27]. These findings suggest several possible pathways—language barriers, limited understanding, contact failure and fragmented information-sharing—but no included study used a validated health-literacy measure or isolated the independent effect of communication support.

Communication should therefore be viewed as a plausible and modifiable contributor rather than an established cause of non-attendance. Future evaluations should test whether communication support improves understanding, reach and sustained attendance.

### 4.6. Service Organisation and the Screening Pathway

Service organisation emerged as a modifiable influence, with recurrent problems in patient contact, information-sharing, integration with diabetes care and variation between practices [16,21,22,23]. In the national Welsh cohort [28], attendance at the first screening invitation was strongly associated with lower subsequent repeat non-attendance, making early engagement a useful service-risk marker rather than a causal explanation.

The Coventry cluster trial [17] provided the only comparative intervention evidence: multilingual link-worker support increased attendance compared to control practices, but reminders, encouragement and language-concordant contact were delivered together and their individual effects could not be separated. Taken together, the service-level evidence supports prioritising modifiable pathway factors rather than treating patient characteristics as causal explanations.

### 4.7. Implications for NHS Screening Practice

The findings should be interpreted within the four separately administered UK screening systems: the NHS Diabetic Eye Screening Programme in England, the nationally coordinated programme in Scotland, Diabetic Eye Screening Wales, and the regional programme in Northern Ireland [29,30,31,32]. Current pathways increasingly use risk-stratified recall, including 24-month recall for people at lowest risk after two consecutive routine screens showing no retinopathy, while annual or more frequent screening, surveillance or hospital pathways remain for those at greater clinical risk [29,30,31,32]. Because much of the included evidence predates these arrangements, inequalities should be monitored across each stage of the contemporary pathway rather than inferred from overall programme uptake alone.

The evidence supports a proportionate approach to improving access. Clear invitations, straightforward rebooking and reminders may benefit all participants, while repeated non-attendance or delayed first screening may justify additional contact verification, telephone outreach, professional interpretation and coordination with primary or diabetes care [33,34,35,36,37]. The qualitative case study and Coventry cluster trial [16,17] support proactive contact, while the national Welsh cohort [28] identifies early attendance and repeated non-attendance as useful markers for further follow-up. For younger adults, the Northern Ireland youth study [20] and Welsh repeat non-attendance cohort [28] support evaluating flexible scheduling, easier rebooking and better coordination with diabetes appointments.

Equity monitoring should therefore combine attendance with clinical risk and more detailed information on ethnicity, preferred language, deprivation, age, residential stability and previous attendance. Ethnicity alone should not determine intervention intensity; resources should instead be directed towards barriers demonstrated within local populations and evaluated for their effect on sustained attendance and inequality reduction.

### 4.8. Strengths and Limitations

A key strength of this review is its focus on organised UK screening pathways while integrating quantitative, qualitative, mixed-methods, professional survey and intervention evidence. This allowed attendance inequalities, possible explanations and service responses to be considered together without treating different evidence types as equivalent. Design-specific quality and risk-of-bias appraisal was undertaken for all included studies, with detailed assessments presented in the Appendix A and an overall visual summary in Figure 2. Appraisal findings informed the weight given to individual findings rather than determining study eligibility. Potentially overlapping datasets, particularly those reported by Lawrenson et al. [18] and Olvera–Barrios et al. [19], were not interpreted as independent confirmation. The updated search, independent dual screening and appraisal, checked data extraction, study-specific full-text exclusions and structured confidence assessment further strengthened methodological transparency.

Several limitations remain. The evidence base was heterogeneous in design, population, exposure and outcome, preventing meta-analysis and limiting causal inference. Eleven studies were England-specific, one was from Northern Ireland, one was a national Welsh patient-level study and one was a UK-wide professional survey; no eligible patient-level study conducted solely in Scotland was identified. Much of the evidence also predates current risk-stratified recall pathways, and no eligible UK primary study published after 2021 was identified in the updated search. Individual studies had further limitations, including broad or incomplete ethnicity data, area- or practice-level measures of deprivation, non-response in questionnaire studies, small qualitative samples and the ten-cluster design of the Bush et al. intervention trial [17]. These limitations reduce precision and generalisability even where findings were directionally consistent.

A formal GRADE assessment was not applied because the review combined heterogeneous observational, qualitative, mixed-methods and intervention evidence and did not generate pooled outcome estimates. Instead, confidence in the principal findings was assessed narratively using methodological limitations, directness, consistency, precision and independence of datasets, as reported in Table 8. This approach improves transparency but does not provide the same outcome-specific certainty grading as a formal GRADE assessment.

The review was not prospectively registered because registration was inadvertently overlooked and review activities had already commenced when this was recognised. This limits external assurance that all methods and decisions were prespecified and increases the possibility of post hoc methodological changes. To mitigate this limitation, the revised review reports the eligibility criteria, complete search strategies, screening procedures, study-specific full-text exclusions, design-specific appraisals and synthesis methods transparently, with independent dual screening and appraisal and checked data extraction.

The review was restricted to UK studies published in English between 2006 and 18 August 2026. This restriction was intended to maintain relevance to contemporary UK diabetic eye screening pathways and NHS service delivery; however, it may have excluded relevant evidence published before 2006, studies conducted in other healthcare systems that could provide transferable insights, and non-English-language research. The geographical, language and date restrictions may therefore have introduced selection or language bias and may limit the generalisability of the findings beyond the UK context. Google Scholar screening was limited to the first five pages of each query, and dedicated conference proceedings, thesis and broad grey-literature databases were not searched. Publication bias could not be formally assessed because the 14 included studies were too heterogeneous for quantitative synthesis. These limitations should be considered when interpreting the findings, although the broadened database search, citation searching and transparent reporting reduce some of the associated uncertainty.

### 4.9. Future Research Priorities

Future research should move beyond broad demographic associations and examine the mechanisms underlying unequal attendance. Linked screening, primary-care and diabetes datasets should include more granular information on ethnicity, preferred language, deprivation, occupation, residential mobility, diabetes type and duration, glycaemic control, previous retinopathy and attendance history. Analyses should distinguish confounding, effect modification and mediation so that differences are not attributed to ethnicity or age when they may instead reflect socioeconomic circumstances, language or service access.

Qualitative research should purposively include persistent non-attenders and under-represented communities rather than relying mainly on people already engaged with services. Younger adults also warrant focused study of employment, education, transitions in diabetes care, housing mobility and other life-stage pressures identified or suggested by Cushley et al. [20], Strutton et al. [21], Prothero et al. [23], Orton et al. [27] and Thomas et al. [28].

Intervention research remains particularly limited. The Coventry cluster trial [17] provides proof-of-concept for proactive multilingual link-worker support, but larger multicentre trials are needed to determine which components are effective and for whom. Future trials should test the access strategies highlighted above—including flexible access, contact verification, language-concordant communication, outreach and integration with diabetes care—and report intervention reach, sustained attendance and subgroup effects. Success should be judged not only by overall uptake but by whether inequalities in attendance are reduced.

## 5. Conclusions

Across the included UK studies, younger age and socioeconomic deprivation were the most consistent factors associated with lower, delayed or repeated non-attendance at diabetic eye screening, whereas ethnicity-related associations varied between groups and settings. Communication difficulties, competing commitments, residential instability, inflexible appointment systems and limited integration with diabetes care also emerged as plausible contributors to unequal participation, although the evidence for specific mechanisms remains limited.

Overall, the findings support a more targeted approach to screening inequalities that focuses on locally demonstrated barriers rather than broad assumptions about demographic groups. Equity monitoring should consider attendance alongside clinical risk and relevant sociodemographic factors, while future interventions should be evaluated for their ability to improve sustained participation and reduce inequalities.

## Figures and Tables

**Table 1:** Population–Interest–Context (PICo) framework for the review question.

Framework Element		Description
P	Population	People with diabetes who are eligible for diabetic eye screening in the UK, with a focus on minority ethnic and other inequality-affected populations.
I	Interest	Attendance-related associated factors, barriers, facilitators and interventions, including ethnicity, language, culture, deprivation, age, access, communication, reminders and service organisation.
Co	Context	UK diabetic eye screening services, including organised NHS screening programmes.

**Table 2:** Eligibility criteria.

Criterion	Include	Exclude
Country/setting	United Kingdom-based diabetic eye screening or diabetic retinopathy screening services	Non-United Kingdom studies or studies without clear United Kingdom relevance
Population	People with diabetes eligible for screening, including minority ethnic or inequality-affected populations	Studies not involving diabetic eye screening populations
Focus	Attendance, uptake or non-attendance and at least one eligible associated factor, barrier, facilitator, access issue, patient/service experience or intervention	No eligible attendance-related associated factor, barrier, facilitator or intervention; diagnostic accuracy, grading, artificial intelligence or disease prevalence only
Study design	Primary qualitative, quantitative, mixed-methods, observational or intervention studies	Reviews, editorials, commentaries and secondary evidence for final synthesis
Language/date	English language, 2006–2026	Outside date range or non-English where no translation was available

**Table 3:** Search and source summary.

Source	Records	Role in Review
Embase via Ovid	520	Expanded database search rerun 18 August 2026
MEDLINE via Ovid	176	Expanded database search rerun 18 August 2026
Web of Science Core Collection	114	Expanded database search rerun 18 August 2026
Scopus	144	Expanded database search rerun 18 August 2026
CENTRAL	31	Expanded database search rerun 18 August 2026
Google Scholar	15	Potentially relevant records from first five pages of six targeted searches
Backward/forward citation searching	12	Additional records identified from included studies and prior reviews
Official UK programme sources	10; excluded from PRISMA count	Policy and programme context only
Total before deduplication	1012	1000 database/Google Scholar records plus 12 citation-search records

**Table 4:** PRISMA 2020 numerical summary. Official UK programme documents were retained for background and policy context only and were not included in the PRISMA record count or primary-study synthesis.

PRISMA Stage	Number
Records identified from Embase via Ovid	520
Records identified from MEDLINE via Ovid	176
Records identified from Web of Science	114
Records identified from Scopus	144
Records identified from CENTRAL	31
Records selected from Google Scholar	15
Records identified through citation searching	12
Total records before deduplication	1012
Duplicate records removed	429
Records screened by title and abstract	583
Records excluded at title/abstract stage	535
Full-text reports sought for retrieval	48
Full-text reports not retrieved	0
Full-text reports assessed for eligibility	48
Full-text reports excluded	34
No ethnicity or inequality focus	5
No eligible attendance-related factor, barrier, facilitator or intervention	16
Secondary evidence	4
Wrong population or location	9
Primary studies included in narrative synthesis	14
Official UK programme documents retained for context only	10

**Table 5:** Characteristics of included primary studies.

Study (Author, Year)	Country	Design and Setting	Population/Sample	Equity Dimension(s) Examined	Main Findings Relevant to Attendance
Scanlon et al., 2008 [15]	England	Cross-sectional observational study; Gloucestershire diabetic eye screening database	13,284 people with diabetes (dataset 1) 10,312 screened (dataset 2)	Socioeconomic deprivation; small ethnic minority population	Screening uptake decreased from 76.7% in the least deprived quintile to 67.4% in the most deprived quintile. Sight-threatening DR was more common in more deprived groups.
Lindenmeyer et al., 2014 [16]	England	Qualitative comparative case study; nine general practices across three screening programme areas	62 participants: 38 patients and 24 professionals including practice staff and screeners	Deprivation; ethnic and language diversity; transport/access	Eight factors influenced uptake, including communication with screening services, patient contact, integration with diabetes care, newly diagnosed patient processes, deprivation, language/culture and transport access.
Bush et al., 2014 [17]	England	Cluster-randomised controlled proof-of-concept trial; 10 general practices in Coventry with high South Asian populations	2680 patients invited	South Asian population; multilingual link workers	Mean practice-level attendance was 89% in intervention practices and 74% in controls (adjusted difference 12 percentage points, 95% confidence interval 7–17). Language-appropriate link-worker calls were promising, while inaccurate contact details limited reach.
Lawrenson et al., 2021 [18]	England	Retrospective cohort; three urban English screening programmes plus vision impairment certification analysis	Cohort A: 97,048 newly registered participants; Cohort B: 291,296 eligible participants	Ethnicity, age, deprivation and urban diversity	Younger age, deprivation, ethnicity and diabetes duration were associated with non-attendance and/or referable DR. Young adults had suboptimal screening uptake despite high overall attendance.
Olvera–Barrios et al., 2021 [19]	England	12-month retrospective cohort; North East London diabetic eye screening programme	84,449 people with diabetes; 83.4% attended	Large multi-ethnic urban population: South Asian, Black, White British, Chinese and other groups	South Asian, Chinese and other Asian groups had higher adjusted odds of attendance than White British participants. Deprivation, younger age, poorer visual acuity and longer diabetes duration were associated with non-attendance.
Cushley et al., 2021 [20]	Northern Ireland	Mixed-methods study; Northern Ireland diabetic eye screening programme	2330 records; 25 questionnaire respondents	Young people aged 12–26; access and communication inequalities	Age, diabetes duration, diabetes clinic attendance and glycated haemoglobin were associated with engagement. Barriers included inconvenient appointment times, work/school commitments, access problems and poor communication.
Strutton et al., 2016 [21]	England	Qualitative service evaluation using Framework analysis; one South London diabetic eye screening programme	296 never-attenders identified; 258 eligible; reasons obtained for 146 (57%)	Persistent never-attendance; communication, service navigation and practical inequalities	Patient-level explanations included competing commitments, anxiety, disengagement from diabetes care and misinformation. System-level explanations included inaccurate or unshared address, clinical and practical information.
Moreton et al., 2017 [22]	England	Analytical cross-sectional study of routine programme data plus optometry appointment survey; Oxfordshire	21,797 invited across 79 general practices; 17,967 attended; 16 optometry practices surveyed	Age, sex, practice-level deprivation, screening modality and appointment availability	Overall attendance was 82.4% and lowest at ages 12–39 years (66.6%). Greater practice-level deprivation was associated with lower attendance. Modality was not independently associated after adjustment; residual between-practice variation remained.
Prothero et al., 2021 [23]	UK	Cross-sectional online survey of healthcare professionals working in diabetic eye screening programmes	580 British Association of Retinal Screening members invited; 140 analysed (24.1%)	Young adults; organisational, access and communication inequalities	Professionals perceived limited integration with diabetes care, access and scheduling difficulties, resource constraints and poor inter-provider communication as barriers. Only 46.4% reported a dedicated young adult strategy; reported effectiveness was perceptual, not an attendance effect.
Gulliford et al., 2010 [24]	England	Cross-sectional analysis of routine screening data; three South London boroughs	31,484 people; 59,495 screening records	Ethnicity, socioeconomic deprivation and age	Seventy-eight per cent received at least one screen. Greater deprivation and younger age were associated with non-attendance; ethnicity was modelled directly, while disease outcomes were analysed separately.
Millett and Dodhia, 2006 [25]	England	Cross-sectional health-equity audit of routine screening data; South East London	8066 participants with electronic records	Age, socioeconomic deprivation and region of birth	Overall attendance was 88.9%. Younger age and greatest area deprivation were independently associated with lower attendance; region of birth was not significantly associated with attendance.
Ahmad and Neilson, 2019 [26]	England	Retrospective health-equity audit of six screening programmes in Cumbria and North East England	196,275 registered people aged 12 years or older	Age, socioeconomic deprivation, sex and geography	Twelve-month uptake ranged from 77% to 84%, but was below 70% among people aged 19–44 years in all six programmes and was lower in more deprived groups. Ethnicity could not be analysed reliably because of incomplete data.
Orton et al., 2013 [27]	England	Mixed-methods health-equity audit; Derbyshire screening programme	1000 questionnaire recipients (43% response); 32 non-attenders interviewed; routine programme data also analysed	Socioeconomic deprivation, age, communication and understanding	Greater deprivation and younger age were independently associated with non-uptake. Patient-reported findings indicated gaps in discussion and understanding of screening.
Thomas et al., 2021 [28]	Wales	Retrospective longitudinal study using linked national screening, primary- and secondary-care records	6513 people with T1DM; 156,525 with T2DM	Age, socioeconomic deprivation, residential mobility and repeat non-attendance	Repeat non-attendance occurred in 18% and 8% of the type 1 and T2DM cohorts. Younger age, greater deprivation and more frequent residential moves were independently associated with repeat non-attendance; first-invitation attendance was strongly associated with lower subsequent repeat non-attendance.

**Table 6:** Summary of quality appraisal and risk-of-bias assessment.

Study	Design	Appraisal Tool	Overall Appraisal	Main Methodological Considerations
Scanlon et al., 2008 [15]	Cross-sectional observational	JBI analytical cross-sectional	Some methodological concerns	Older data; area-level deprivation; limited ethnicity and clinical confounders.
Lindenmeyer et al., 2014 [16]	Qualitative case study	JBI qualitative	Some methodological concerns	Limited reflexive reporting and transferability beyond sampled practices.
Bush et al., 2014 [17]	Cluster-randomised trial	Cochrane RoB 2 for cluster-randomised trials	Some concerns	Ten clusters; possible baseline imbalance; aggregate effects; reach depended on successful contact.
Lawrenson et al., 2021 [18]	Retrospective cohort	NOS	8/9 stars; comparatively lower concern	Observational; missing ethnicity and clinical data; cannot explain mechanisms.
Olvera–Barrios et al., 2021 [19]	Retrospective cohort	NOS	7/9 stars; some concerns	Single regional programme; observational; partly overlaps geographically with Lawrenson et al.
Cushley et al., 2021 [20]	Mixed-methods	JBI split appraisal	Some methodological concerns	Small questionnaire/free-text sample; persistent non-attenders under-represented.
Strutton et al., 2016 [21]	Qualitative service evaluation	JBI qualitative	Some methodological concerns	Reasons obtained for 57%; non-verbatim staff notes; limited governance reporting.
Moreton et al., 2017 [22]	Analytical cross-sectional	JBI analytical cross-sectional	Comparatively lower concern	Practice-postcode deprivation proxy; no ethnicity, blood-pressure or glycaemic data.
Prothero et al., 2021 [23]	Cross-sectional professional survey	Adapted JBI prevalence	Some methodological concerns	24.1% response; no sample-size rationale; possible non-response bias; perceived rather than measured effects.
Gulliford et al., 2010 [24]	Cross-sectional routine-data analysis	JBI analytical cross-sectional	Comparatively lower concern	Large adjusted routine dataset; observational design; missing or broad demographic data limit causal interpretation.
Millett and Dodhia, 2006 [25]	Cross-sectional health-equity audit	JBI analytical cross-sectional	Some methodological concerns	Electronic records available for 82.7% of referred participants; incomplete coding, limited ethnicity data and area-level deprivation.
Ahmad and Neilson, 2019 [26]	Retrospective health-equity audit	JBI analytical cross-sectional	Some methodological concerns	Very large multi-programme audit, but primarily descriptive; no multivariable adjustment; incomplete ethnicity and sex data; area-level deprivation.
Orton et al., 2013 [27]	Mixed-methods health-equity audit	JBI analytical cross-sectional for quantitative component	Some methodological concerns	Adjusted routine-data analysis, but 43% questionnaire response and only 32 non-attenders interviewed; residual confounding possible.
Thomas et al., 2021 [28]	Retrospective longitudinal cohort	NOS	8/9; comparatively lower concern	National-linked cohort with objective attendance records and multivariable adjustment; missing clinical covariates, area-level deprivation and model-calibration limitations.

**Table 7:** Summary of barriers and facilitators identified in the included studies.

Theme	Findings from Included Studies	Reported or Evaluated Facilitators/Service Responses	Supporting Studies
Ethnicity and language	Attendance associations varied by ethnic group and setting. Linguistic diversity, missing ethnicity data, misinformation and incomplete contact information complicated interpretation and navigation.	Multilingual link-worker contact was evaluated as a combined reminder, encouragement and language-concordant intervention; clearer and language-appropriate communication was proposed.	Lindenmeyer [16]; Bush [17]; Lawrenson [18]; Olvera–Barrios [19]; Strutton [21]; Gulliford [24]; Millett and Dodhia [25]
Socioeconomic deprivation	Lower attendance was reported across multiple English studies and in the Welsh repeat non-attendance cohort; one smaller Northern Irish youth study found no significant association.	Local assessment of practical and organisational barriers was proposed; no intervention has directly tested reduction in a deprivation-related attendance gap.	Scanlon [15]; Lawrenson [18]; Olvera–Barrios [19]; Cushley [20]; Moreton [22]; Gulliford [24]; Millett and Dodhia [25]; Ahmad and Neilson [26]; Orton [27]; Thomas [28]
Younger age and life stage	Younger people had lower, delayed or repeatedly missed attendance across several independent datasets; work, education and organisational barriers were reported or perceived.	Flexible appointments, straightforward rebooking, reminders and coordination with diabetes care were proposed, but have not been comparatively evaluated specifically for younger adults.	Lawrenson [18]; Olvera–Barrios [19]; Cushley [20]; Moreton [22]; Prothero [23]; Gulliford [24]; Millett and Dodhia [25]; Ahmad and Neilson [26]; Orton [27]; Thomas [28]
Access and appointment logistics	Transport, competing commitments, inflexible booking, variable appointment availability, residential mobility and rescheduling difficulties impeded or were perceived to impede access.	Flexible booking, community delivery and contact verification were proposed; screening modality showed no independent association after adjustment in one study.	Lindenmeyer [16]; Cushley [20]; Strutton [21]; Moreton [22]; Prothero [23]; Orton [27]; Thomas [28]
Communication and understanding	Language needs, misinformation, inaccurate records, service communication failures and limited understanding of screening were reported.	Clear information, accurate records, professional interpretation and provider information-sharing were proposed; no communication format was independently evaluated.	Lindenmeyer [16]; Cushley [20]; Strutton [21]; Prothero [23]; Orton [27]
Service integration and proactive contact	Fragmented practice-screening communication, limited integration with diabetes care, inaccurate contact information and repeated non-attendance constrained delivery and reach.	Multilingual link-worker support improved attendance versus control in one trial; integrated care, early follow-up and reminders were otherwise proposed or supported by observational evidence.	Lindenmeyer [16]; Bush [17]; Strutton [21]; Moreton [22]; Prothero [23]; Thomas [28]

**Table 8:** Narrative confidence in the principal findings. These transparent judgements are not formal GRADE ratings.

Principal Finding	Narrative Confidence	Basis for Judgement
Younger age is associated with lower or delayed attendance	Moderate-to-high	Consistent direction across nine quantitative studies and several independent settings [18,19,20,22,24,25,26,27,28], including large adjusted cohorts; reduced by observational designs and heterogeneous attendance definitions.
Socioeconomic deprivation is associated with lower attendance	Moderate-to-high	Consistent association across multiple independent English analyses and the Welsh cohort [15,18,19,22,24,25,26,27,28]; reduced by area- or practice-level proxies, residual confounding and one null youth study [20].
Ethnicity-related associations vary by group and setting	Low-to-moderate	Adjusted cohorts and South London analyses support heterogeneity [18,19,24,25], but broad categories, incomplete ethnicity recording and partial cohort overlap limit precision and national generalisation.
Communication, access and practical barriers may reduce attendance	Low-to-moderate	Themes recur across qualitative, mixed-methods, survey and health-equity evidence [16,20,21,23,27,28], but mechanisms were not independently quantified and some samples were small or incomplete.
Service integration and proactive contact may facilitate attendance	Low	Supported by convergent qualitative, routine-data and professional perception evidence [16,21,22,23,28], with few direct comparative evaluations.
Multilingual link-worker support improves attendance versus control	Low	One proof-of-concept cluster trial reported benefit [17], but included ten clusters, aggregate outcomes and a combined intervention whose active component and durability are uncertain.

## Data Availability

All data supporting the findings of this systematic review are contained within the article, Appendix A and the Supplementary Materials. No new primary participant data were generated.

## Supplementary Materials

The following supporting information can be downloaded at https://www.mdpi.com/article/10.3390/vision10040066/s1; Table S1: Completed Preferred Reporting Items for Systematic Reviews and Meta-Analyses 2020 checklist [11]; Table S2: Full-text reports excluded after eligibility assessment, with study-specific reasons (n = 34).

## Abbreviations

The following abbreviations are used in this manuscript:

CENTRAL	Cochrane Central Register of Controlled Trials
CI	Confidence interval
DM	Diabetes mellitus
DR	Diabetic retinopathy
GRADE	Grading of Recommendations Assessment, Development and Evaluation
JBI	Joanna Briggs Institute
NHS	National Health Service
NOS	Newcastle–Ottawa Scale
OR	Odds ratio
PICo	Population–Interest–Context
PICO	Population–Intervention/Interest–Comparator–Outcome
PRISMA	Preferred Reporting Items for Systematic Reviews and Meta-Analyses
RoB 2	Risk of Bias 2
T1DM	Type 1 diabetes mellitus
T2DM	Type 2 diabetes mellitus
UK	United Kingdom

## Appendix A

**Table A1:** Search strategies.

Database/Source	Search Strategy
MEDLINE via Ovid	1 exp Diabetic Retinopathy/; 2 exp Mass Screening/or exp Vision Screening/; 3 (diabet* adj3 (eye or retin* or retina* or fundus) adj3 screen*).ti,ab,kf.; 4 ((diabetic eye or diabetic retinopathy or diabetes retinal) adj2 screen*).ti,ab,kf.; 5 1 and (2 or screen*.ti,ab,kf.); 6 3 or 4 or 5; 7 exp Patient Compliance/or exp Health Services Accessibility/or Appointments and Schedules/or exp Age Factors/or exp Socioeconomic Factors/or exp Ethnicity/; 8 (attend* or nonattend* or non-attend* or uptake or participation or engagement or access* or barrier* or facilitator* or enabler* or reminder* or outreach or inequ* or equit* or disparit* or ethnic* or minorit* or deprivation or socioeconomic or socio-economic or poverty or age or young adult* or adolescen* or language* or cultur* or migrant* or mental illness or disabilit*).ti,ab,kf.; 9 7 or 8; 10 exp United Kingdom/or (United Kingdom or UK or England or Scotland or Wales or Northern Ireland or NHS or British).ti,ab,ad.; 11 6 and 9 and 10; 12 limit 11 to English language; 13 limit 12 to yr = “2006-Current”.
Embase via Ovid	1 exp diabetic retinopathy/; 2 exp mass screening/or exp vision screening/; 3 (diabet* adj3 (eye or retin* or retina* or fundus) adj3 screen*).ti,ab,kw.; 4 ((diabetic eye or diabetic retinopathy or diabetes retinal) adj2 screen*).ti,ab,kw.; 5 1 and (2 or screen*.ti,ab,kw.); 6 3 or 4 or 5; 7 exp patient compliance/or exp health care access/or exp appointment/or exp age/or exp socioeconomic status/or exp ethnicity/; 8 (attend* or nonattend* or non-attend* or uptake or participation or engagement or access* or barrier* or facilitator* or enabler* or reminder* or outreach or inequ* or equit* or disparit* or ethnic* or minorit* or deprivation or socioeconomic or socio-economic or poverty or age or young adult* or adolescen* or language* or cultur* or migrant* or mental illness or disabilit*).ti,ab,kw.; 9 7 or 8; 10 exp United Kingdom/or (United Kingdom or UK or England or Scotland or Wales or Northern Ireland or NHS or British).ti,ab,ad.; 11 6 and 9 and 10; 12 limit 11 to English language; 13 limit 12 to yr = “2006-Current”.
Web of Science Core Collection	TS = ((diabet* NEAR/3 (eye OR retin* OR retina* OR fundus) NEAR/3 screen*) AND (attend* OR nonattend* OR “non-attendance” OR uptake OR participation OR engagement OR access* OR barrier* OR facilitator* OR enabler* OR reminder* OR outreach OR inequ* OR equit* OR disparit* OR ethnic* OR minorit* OR deprivation OR socioeconomic OR “socio-economic” OR poverty OR age OR “young adult*” OR adolescen* OR language* OR cultur* OR migrant* OR “mental illness” OR disabilit*) AND (“United Kingdom” OR UK OR England OR Scotland OR Wales OR “Northern Ireland” OR NHS OR British)); filters: English; 2006–2026.
Scopus	TITLE-ABS-KEY((diabet* W/3 (eye OR retin* OR retina* OR fundus) W/3 screen*) AND (attend* OR nonattend* OR “non-attendance” OR uptake OR participation OR engagement OR access* OR barrier* OR facilitator* OR enabler* OR reminder* OR outreach OR inequ* OR equit* OR disparit* OR ethnic* OR minorit* OR deprivation OR socioeconomic OR “socio-economic” OR poverty OR age OR “young adult*” OR adolescen* OR language* OR cultur* OR migrant* OR “mental illness” OR disabilit*) AND (“United Kingdom” OR UK OR England OR Scotland OR Wales OR “Northern Ireland” OR NHS OR British)) AND PUBYEAR > 2005 AND PUBYEAR < 2027 AND (LIMIT-TO(LANGUAGE, “English”)).
Cochrane Central Register of Controlled Trials	(([mh “Diabetic Retinopathy”] AND ([mh “Mass Screening”] OR [mh “Vision Screening”] OR screen*:ti,ab,kw)) OR (diabet* NEAR/3 (eye OR retin* OR retina* OR fundus) NEAR/3 screen*):ti,ab,kw) AND (attend* OR nonattend* OR “non-attendance” OR uptake OR participation OR engagement OR access* OR barrier* OR facilitator* OR enabler* OR reminder* OR outreach OR inequ* OR equit* OR disparit* OR ethnic* OR minorit* OR deprivation OR socioeconomic OR “socio-economic” OR poverty OR age OR “young adult*” OR adolescen* OR language* OR cultur* OR migrant* OR “mental illness” OR disabilit*):ti,ab,kw AND ([mh “United Kingdom”] OR “United Kingdom” OR UK OR England OR Scotland OR Wales OR “Northern Ireland” OR NHS OR British):ti,ab,kw; publication years 2006–2026.
Google Scholar	Six targeted searches: (1) “diabetic eye screening” UK ethnicity barriers attendance; (2) “diabetic retinopathy screening” UK “ethnic minority” non-attendance; (3) “diabetic eye screening” UK qualitative barriers uptake; (4) “diabetic eye screening” UK “South Asian”; (5) “diabetic eye screening” UK age “young adults” attendance; (6) “diabetic eye screening” UK deprivation socioeconomic uptake. The first five pages were screened for each search.
Citation searching	Backward reference-list and forward cited-by searching of all included studies and the two earlier systematic reviews; 12 additional records were exported for screening.
Official UK programme sources	Targeted searches used terms relating to diabetic eye screening, ethnicity, inequalities, language, non-attendance and health equity. Documents were used for context only.

Note: Searches were limited to English-language records published from 2006 to 18 August 2026. Controlled vocabulary and free-text terms were combined in databases that supported subject headings. Grey-literature documents were retained for context only.

**Table A2:** Adapted the Population–Intervention/Interest–Comparator–Outcome (PICO) eligibility framework.

Framework Element	Description for This Review
Population	People with diabetes eligible for diabetic eye screening in the UK, with a focus on minority ethnic communities and inequality-affected groups, including those affected by language, culture, deprivation or access barriers.
Intervention/Interest	Factors influencing diabetic eye screening attendance, including barriers, facilitators, patient experiences, service-level factors, communication, reminders, link workers, health literacy and culturally appropriate support.
Comparator	Not applicable for the main review question. Where relevant, studies comparing attenders and non-attenders, intervention and control groups, or different demographic groups were included.
Outcome	Attendance, uptake or non-attendance at diabetic eye screening, alongside reported barriers, facilitators and recommendations to improve equitable access.

**Table A3:** NOS appraisal for retrospective cohort studies.

NOS Domain	Lawrenson et al. [18]	Olvera–Barrios et al. [19]	Thomas et al. [28]
Representativeness of cohort	Yes—large routine screening programme cohort from three urban English diabetic eye screening programmes.	Yes—large routine screening programme cohort from North East London diabetic eye screening programme.	Yes—national Welsh diabetic eye screening cohort with linked primary- and secondary-care records.
Selection of comparison group	Yes—comparison groups were drawn from the same screening programme populations.	Yes—comparison groups were drawn from the same regional screening programme population.	Yes—repeat non-attenders and attenders were drawn from the same screening population.
Ascertainment of exposure	Yes—demographic and clinical exposures were obtained from routine screening programme records.	Yes—ethnicity, deprivation and sociodemographic data were obtained from screening programme records.	Yes—demographic, deprivation, residential-mobility and clinical variables were obtained from linked routine records.
Outcome not present at start of study	Yes/Partly—attendance outcomes were assessed using screening records, although the study was observational and retrospective.	Yes—attendance over the 12-month study period was assessed using programme data.	Partly—repeat non-attendance was identified retrospectively across screening histories; temporality is appropriate for follow-up but the design is observational.
Comparability of groups	Yes—analyses adjusted for relevant demographic and clinical variables.	Yes—analyses adjusted for relevant variables including age, ethnicity and deprivation.	Yes—models adjusted for relevant demographic, socioeconomic and clinical variables.
Assessment of outcome	Yes—screening attendance was measured using routine programme data.	Yes—attendance was measured using routine diabetic eye screening programme data.	Yes—attendance was measured from national screening records.
Follow-up long enough for outcome to occur	Yes—follow-up was sufficient to assess screening attendance and programme outcomes.	Yes—12-month follow-up was sufficient to assess attendance.	Yes—a 36-month definition captured three annual invitation cycles.
Adequacy of follow-up	Yes/Partly—large cohort, but some missing ethnicity, diabetes type and duration data.	Partly—large cohort, but findings were limited to one regional programme.	Partly—large linked cohort, but inclusion required adequate screening history and some clinical covariates had substantial missingness.
Selection score	3/4	3/4	3/4
Comparability score	2/2	2/2	2/2
Outcome score	3/3	2/3	3/3
Total NOS score	8/9	7/9	8/9
Overall judgement	High methodological quality.	Moderate-to-high methodological quality.	High methodological quality/comparatively lower concern.
Key limitations	Observational design; missing ethnicity/type/duration data; unable to explain patient-level reasons for non-attendance.	Observational design; single regional programme; unable to establish causality or explain individual reasons for non-attendance.	Retrospective design; area-level deprivation; substantial missingness for some clinical covariates; model-calibration limitations; causal mechanisms cannot be established.

**Table A4:** JBI analytical cross-sectional appraisal. Items: 1, inclusion criteria; 2, participants and setting; 3, exposure measurement; 4, objective outcome criteria; 5, confounders identified; 6, strategies addressing confounding; 7, outcome measurement; 8, statistical analysis.

Study	Item-Level Judgements (Items 1–8)	Overall Interpretation	Key Concerns
Scanlon et al. [15]	1 Yes; 2 Yes; 3 Yes; 4 Yes; 5 Unclear; 6 Unclear; 7 Yes; 8 Yes	Some methodological concerns	Area-level deprivation; limited ethnicity and clinical confounding data.
Moreton et al. [22]	1 Yes; 2 Yes; 3 Yes; 4 Yes; 5 Yes; 6 Yes; 7 Yes; 8 Yes	Comparatively lower concern	Practice-level deprivation proxy; residual confounding.
Cushley et al. [20], quantitative component	1 Yes; 2 Yes; 3 Unclear; 4 Yes; 5 Unclear; 6 Unclear; 7 Yes; 8 Yes	Some methodological concerns	Small questionnaire component; limited confounding control.
Gulliford et al. [24]	1 Yes; 2 Yes; 3 Yes; 4 Yes; 5 Yes; 6 Yes; 7 Yes; 8 Yes	Comparatively lower concern	Large adjusted routine-data analysis, but observational; missing demographic or clinical data and broad ethnic categories.
Millett and Dodhia [25]	1 Yes; 2 Yes; 3 Partly; 4 Yes; 5 Yes; 6 Yes; 7 Yes; 8 Yes	Some methodological concerns	Electronic records available for 82.7% of referred participants; incomplete coding; area-level deprivation; ethnicity not directly analysable.
Ahmad and Neilson [26]	1 Yes; 2 Yes; 3 Partly; 4 Yes; 5 Yes; 6 No; 7 Yes; 8 Yes	Some methodological concerns	Descriptive health-equity audit without multivariable adjustment; incomplete ethnicity and sex data; area-level deprivation.
Orton et al. [27], quantitative component	1 Yes; 2 Yes; 3 Yes; 4 Yes; 5 Yes; 6 Yes; 7 Yes; 8 Yes	Some methodological concerns overall	Adjusted routine-data analysis; overall mixed-methods study limited by 43% questionnaire response, small non-attender interview subsample and residual confounding.

**Table A5:** JBI qualitative appraisal.

JBI Qualitative Item	Lindenmeyer et al. [16]	Strutton et al. [21]	Cushley et al. [20]: Qualitative/Free-Text Component
1. Congruity: philosophical perspective and methodology	Unclear	Unclear	Unclear
2. Congruity: methodology and research question	Yes	Yes	Yes
3. Congruity: methodology and data collection	Yes	Yes	Yes
4. Congruity: methodology and data representation/analysis	Yes	Yes	Unclear
5. Congruity: methodology and interpretation	Yes	Yes	Yes
6. Researcher located culturally/theoretically	No	No	No
7. Researcher influence addressed	Unclear	Yes	No
8. Participant voices adequately represented	Yes	Yes	Unclear
9. Ethical conduct/approval reported	Yes	Unclear	Yes
10. Conclusions flow from analysis	Yes	Yes	Yes
Overall interpretation	Some methodological concerns	Some methodological concerns	Some methodological concerns
Key concerns	Limited reflexivity and transferability	Non-verbatim notes; limited governance reporting	Limited qualitative depth and reflexivity

**Table A6:** Adapted JBI appraisal for the cross-sectional professional survey.

JBI Prevalence Item	Prothero et al. [23]
1. Sample frame appropriate	Yes
2. Participants sampled appropriately	Yes
3. Adequate sample size	No
4. Subjects and setting described in detail	Yes
5. Sufficient coverage in data analysis	Yes
6. Valid methods for identifying reported beliefs	Yes
7. Standard and reliable measurement for all participants	Yes
8. Appropriate statistical analysis	Yes
9. Adequate response rate or management of low response	No
Overall interpretation	Some methodological concerns
Key concerns	24.1% response; no sample-size rationale; possible non-response bias; professional perceptions rather than observed attendance effects

**Table A7:** Cochrane RoB 2 appraisal for the cluster-randomised trial.

Cochrane RoB 2 Domain	Bush et al. [17]
Bias arising from the randomisation process	Some concerns—general practices were cluster-randomised, but the number of clusters was small and baseline imbalance between practices was possible.
Bias arising from timing of identification or recruitment of participants	Some concerns—the intervention was delivered at practice/cluster level and individual-level recruitment processes were not fully detailed.
Bias due to deviations from intended interventions	Some concerns—the intervention was pragmatic and depended on whether link-worker telephone contact was successfully achieved.
Bias due to missing outcome data	Some concerns—attendance outcomes were available, but the intervention effect varied according to whether patients were successfully contacted.
Bias in measurement of the outcome	Low risk—attendance at diabetic retinopathy screening was an objective programme outcome.
Bias in selection of the reported result	Some concerns—this was a proof-of-concept study with limited reporting detail.
Overall risk-of-bias judgement	Some concerns.
Key limitations	Proof-of-concept design; small number of general practice clusters; possible baseline imbalance; aggregate-level data; intervention effect depended on successful patient contact.

**Table A8:** Grey-literature sources retained for background and policy context.

Document	Organisation	Source Type	Use in Review	Included in Primary-Study Synthesis?
NHS diabetic eye screening programme collection [38]	NHS England/GOV.UK	Programme hub	Used to identify official National Health Service diabetic eye screening programme resources.	No
Diabetic eye screening: programme overview [3]	NHS England/GOV.UK	Programme overview	Used in the Introduction to describe the programme, target population and aim of screening.	No
Diabetic eye screening: professional guidance [36]	NHS England/GOV.UK	Professional guidance collection	Used in the Discussion to support service delivery and communication recommendations.	No
Diabetic eye screening: information leaflets [33]	NHS England/GOV.UK	Patient information	Used to support the discussion of language, accessibility, patient understanding and easy-read resources.	No
Diabetic eye screening programme standards [35]	NHS England/GOV.UK	Programme standards	Used to provide background on quality standards and service expectations.	No
Diabetic eye screening: programme-specific operating model [37]	NHS England/GOV.UK	Quality assurance guidance	Used to support the discussion of quality assurance, service improvement and programme delivery.	No
Diabetic eye screening intervals extended for people at lowest risk [29]	NHS England/GOV.UK	Recall guidance	Used to describe England’s 24-month low-risk recall and more frequent pathways.	No
Diabetic eye screening [30]	NHS National Services Scotland	Programme overview	Used to describe Scottish delivery through National Health Service Boards and recall ranging from 3 to 24 months.	No
Diabetic eye screening [31]	Public Health Wales	Programme overview	Used to describe the national Welsh programme and 24-month low-risk recall.	No
Diabetic eye screening [32]	nidirect/Northern Ireland Executive	Public information	Used to describe Northern Ireland’s eligibility, invitation and low-risk recall pathway.	No
