# Supplementary material for: Diabetic Eye Screening Attendance Among Minority Ethnic and Other Inequality-Affected Populations in the United Kingdom: A Systematic Review of Associated Factors, Barriers, Facilitators and Interventions
Source: Vision (Basel). 2026 Sep 4;10(4):66. doi: 10.3390/vision10040066 (PMC13600017; doi:10.3390/vision10040066)
Supplement: Supplementary file 1 [file vision-10-00066-s001.zip › v8 Suplementary Table S2 FINAL.pdf]

Supplementary Table S2 for Diabetic Eye Screening Attendance among Minority Ethnic and Other Inequality-Affected Populations in the United Kingdom: A Systematic Review of Associated Factors, Barriers, Facilitators and Interventions

Supplementary Table S2. Full-text reports excluded after eligibility assessment, with study-specific reasons (n = 34)

Key to exclusion reasons: A = no ethnicity or inequality focus (n = 5); B = no eligible attendance-related factor, barrier, facilitator or intervention (n = 16); C = secondary evidence (n = 4); D = wrong population or location (n = 9).

No.	Report (Vancouver style)	Study-specific reason for exclusion	Category
1	Geran-Leech J, Rennie C, Krishnan R, Rahman N. Barriers to care: examining socioeconomic determinants and barriers to clinical attendance for diabetic eye disease. Eye. 2025;39:248. doi:10.1038/s41433-025-03831-0.	Focuses on clinical attendance/care for diabetic eye disease rather than attendance at a diabetic eye screening programme or an eligible diabetic eye screening attendance determinant or intervention.	B
2	Muldrew KA, Ambaye YA, Hogg RE, McGuinness B, Peto T, Wright DM. Exploring the relationship between health inequalities and age-related eye diseases in Northern Ireland. Invest Ophthalmol Vis Sci. 2025;66(8).	Examines inequalities in age-related eye diseases in Northern Ireland rather than diabetic eye screening attendance.	B
3	Balafshan T, Sullivan H, Keigan S, Wilson A, Oyedeji I. Revolutionising young adult diabetes care: a patient-centred transformation to improve access, engagement and outcomes in one of England's most deprived areas. Future Healthc J. 2025;12(2):100431. doi:10.1016/j.fhj.2025.100431.	Evaluates engagement with young-adult diabetes care generally; diabetic eye screening attendance is not the eligible attendance outcome.	B
4	Crosby-Nwaobi R, Suthar S, Shek Y, Valentine S, Khan H, Baker H, et al. Promoting ophthalmic research in underserved communities. Invest Ophthalmol Vis Sci. 2024;65(7):2157.	Focuses on participation in ophthalmic research among underserved communities, not diabetic eye screening attendance.	B
5	Spencer LH, Albustami M, Porter A, Naha G, Thomas RL, Rees S, et al. Uptake of diabetes follow-up checks by minority community groups: a rapid review of the evidence for the CYMELL study. Lancet. 2023;402:S86. doi:10.1016/S0140-6736(23)02072-X.	Addresses uptake of diabetes follow-up checks broadly rather than an eligible diabetic eye screening attendance outcome.	B
6	Jones D, Bhakta G, Lewis H. Factors associated with 'did not attend' outcomes in a UK screening programme: an important source of health inequity. J Epidemiol Community Health. 2023;77:A68. doi:10.1136/jech-2023-SSMabstracts.137.	Examines factors associated with non-attendance in a UK screening programme, but does not specifically evaluate minority ethnicity or another prespecified inequality-affected population as the review focus.	A
7	Kashim RM, Newton P, Ojo O. Diabetic retinopathy screening: a systematic review on patients' non-attendance. Int J Environ Res Public Health. 2018;15(1):157.	Systematic review of diabetic retinopathy screening non-attendance; excluded because the review includes primary studies only.	C

No.	Report (Vancouver style)	Study-specific reason for exclusion	Category
8	Piyasena MMPN, Murthy GVS, Yip JL, Gilbert C, Zuurmond M, Peto T, et al. Systematic review on barriers and enablers for access to diabetic retinopathy screening services in different income settings. PLoS One. 2019;14(4):e0198979.	Systematic review of barriers and enablers to diabetic retinopathy screening; excluded because the review includes primary studies only.	C
9	Nugawela MD, Gurudas S, Prevost AT, Mathur R, Robson J, Hanif W, et al. Ethnic disparities in the development of sight-threatening diabetic retinopathy in a UK multi-ethnic population with diabetes: an observational cohort study. J Pers Med. 2021;11(8):740.	Examines ethnic disparities in development of sight-threatening diabetic retinopathy rather than diabetic eye screening attendance.	B
10	Graham-Rowe E, Lorencatto F, Lawrenson J, Burr J, Grimshaw JM, Ivers N, et al. Barriers to and enablers of diabetic retinopathy screening attendance: a systematic review of published and grey literature. Diabet Med. 2018;35(10):1308-1319.	Systematic review of barriers/enablers of diabetic retinopathy screening attendance; excluded because the review includes primary studies only.	C
11	Hamid A, Jacob SJ, Bellary SB, Golar SG. Why do patients default eye clinic appointments after referral from the screening programme? Eur J Ophthalmol. 2019;29(3):NP35. doi:10.1177/1120672119847084.	Outcome is defaulting ophthalmology/eye-clinic appointments after screening referral, not attendance at the diabetic eye screening programme itself.	B
12	Marks D, Holdsworth E, Datta J, Leamon S, Lee H, Macdowall W. The use of a telephone reminder to improve attendance at a diabetic retinopathy outpatient clinic: An evaluation of a pilot intervention in Bradford. Diabet Med. 2015;32:198-199. doi:10.1111/dme.12668_1.	Intervention targets attendance at a diabetic retinopathy outpatient clinic rather than attendance at the diabetic eye screening programme.	B
13	Low L, Ogston S, Wilson P, MacEwen C. Is socioeconomic deprivation associated with prevalence of diabetic retinopathy in a free access national screening programme? Invest Ophthalmol Vis Sci. 2014;55(13):5348.	Examines socioeconomic deprivation in relation to diabetic retinopathy prevalence rather than an eligible screening-attendance outcome.	B
14	Lee H, Appelbee E, Hurrell DL, Leamon S. The barriers and enablers that affect access to diabetic retinopathy screening amongst Pakistani people in Bradford and Glasgow. Diabet Med. 2013;30:138. doi:10.1111/dme.12091_2.	Conference abstract only; insufficient methodological and outcome detail for reliable extraction and critical appraisal.	B
15	Bachmann MO, Eachus J, Hopper CD, Smith GD, Proppert C, Pearson NJ, et al. Socio-economic inequalities in diabetes complications, control, attitudes and health service use: A cross-sectional study. Diabetic Med. 2003;20(11):921-929. doi:10.1046/j.1464-5491.2003.01050.x.	Examines socioeconomic inequalities across diabetes complications, control, attitudes and health-service use rather than diabetic eye screening attendance specifically.	B

No.	Report (Vancouver style)	Study-specific reason for exclusion	Category
16	England L, O'Connor A. Do Socioeconomic Inequalities Exist Within Ophthalmology and Orthoptics in the UK?: A Scoping Review. Br Ir Orthopt J. 2024;20(1):31-47. doi:10.22599/bioj.338.	Scoping review of socioeconomic inequalities in UK ophthalmology/orthoptics; excluded because the review includes primary studies only.	C
17	Leese G, Boyle P, Feng Z, Emslie-Smith A, Ellis J. Screening Uptake in a Well-Established Diabetic Retinopathy Screening Program The role of geographical access and deprivation. Diabetes Care. 2008;31(11):2131-2135. doi:10.2337/dc08-1098.	Examines geographical access/deprivation and screening uptake, but the report was not retained as an eligible study focused on the prespecified minority-ethnic/inequality population and attendance question.	A
18	Kelly S, Loiselle A, Pandey R, Combes A, Murphy C, Kavanagh H, et al. Factors associated with non-attendance in the Irish national diabetic retinopathy screening programme (INDEAR study report no. 2). Acta Diabetol. 2021;58(5):643-650. doi:10.1007/s00592-021-01671-4.	Study is from the Republic of Ireland rather than the United Kingdom.	D
19	Banks J, Olvera-Barrios A, Pitt M, Williams D, Seltene M, Rutowska C, et al. Effect of ethnicity and other sociodemographic factors on attendance at ophthalmology appointments following referral from a Diabetic Eye Screening Programme: a retrospective cohort study. BMJ Open Ophthalmol. 2025;10(1). doi:10.1136/bmjophth-2024-001969.	Outcome is attendance at ophthalmology appointments after referral from diabetic eye screening, not attendance at diabetic eye screening itself.	B
20	van Eijk K, Blom J, Gussekloo J, Polak B, Groeneveld Y. Diabetic retinopathy screening in patients with diabetes mellitus in primary care: Incentives and barriers to screening attendance. Diabetes Res Clin Pract. 2012;96(1):10-16. doi:10.1016/j.diabres.2011.11.003.	Primary-care diabetic retinopathy screening study conducted outside the United Kingdom.	D
21	Thomas C, Channa R, Prichett L, Liu T, Abramoff M, Wolf R. Racial/Ethnic Disparities and Barriers to Diabetic Retinopathy Screening in Youths. JAMA Ophthalmol. 2021;139(7):791-795. doi:10.1001/jamaophthalmol.2021.1551.	Study population is outside the United Kingdom.	D
22	Fairless E, Nwanyanwu K. Barriers to and Facilitators of Diabetic Retinopathy Screening Utilization in a High-Risk Population. J Racial Ethnic Health Disparities. 2019;6(6):1244-1249. doi:10.1007/s40615-019-00627-3.	Study population is outside the United Kingdom.	D
23	Mc Hugh S, Buckley C, Murphy K, Doherty S, O'Keeffe G, Alade J, et al. Quality-assured screening for diabetic retinopathy delivered in primary care in Ireland: an observational study. Br J Gen Pract. 2013;63(607):E134-E140. doi:10.3399/bjgp13X663091.	Study is from the Republic of Ireland rather than the United Kingdom.	D

No.	Report (Vancouver style)	Study-specific reason for exclusion	Category
24	Scanlon P, Nevill C, Stratton I, Maruti S, Massó-González E, Sivaprasad S, et al. Prevalence and incidence of diabetic retinopathy (DR) in the UK population of Gloucestershire. Acta Ophthalmol. 2022;100(2):E560-E570. doi:10.1111/aos.14927.	Examines prevalence and incidence of diabetic retinopathy rather than screening attendance or an attendance-related determinant/intervention.	B
25	Petersen G, Byberg S, Vistisen D, Fangel M, Vorum H, Joensen L, et al. Factors Associated With Nonattendance in a Nationwide Screening Program for Diabetic Retinopathy: A Register-Based Cohort Study. Diabetes Care. 2022;45(2):303-310. doi:10.2337/dc21-1380.	Nationwide screening cohort was conducted outside the United Kingdom.	D
26	Judah G, Valabhji J, Gunn L, Tyacke P, Vlaev I, King D, et al. A randomised controlled trial on the impact of financial incentives on attendance at diabetic eye screening in London. Diabetologia. 2016;59(1):S65. doi:10.1007/s00125-016-4046-9.	UK intervention study of financial incentives for screening attendance, but the study is not specifically focused on minority-ethnic or another prespecified inequality-affected population.	A
27	Kale AU, Mills A, Guggenheim E, Gee D, Bodza S, Anumakonda A, et al. A Datasheet for the INSIGHT Birmingham, Solihull, and Black Country Diabetic Retinopathy Screening Dataset. Ophthalmol Sci. 2023;3(3). doi:10.1016/j.xops.2023.100293.	Dataset description/resource paper; does not test an eligible attendance-related factor, barrier, facilitator or intervention.	B
28	Chanda UL, Madasu A, Bhardwaj A, Mohyudin M. Barriers to Yearly Diabetic Retinopathy Screening Attendance: A Retrospective Clinical Audit. Cureus. 2024;16(12):e75474. doi:10.7759/cureus.75474.	United Kingdom primary-care audit of screening non-attendance, but it did not have a substantive minority-ethnic or prespecified inequality-focused population or analysis under the review eligibility framework.	A
29	Riordan F, Murphy A, Dillon C, Browne J, Kearney PM, Smith SM, et al. Feasibility of a multifaceted implementation intervention to improve attendance at diabetic retinopathy screening in primary care in Ireland: a cluster randomised pilot trial. BMJ Open. 2021;11(10):e051951. doi:10.1136/bmjopen-2021-051951.	Intervention study conducted in the Republic of Ireland rather than the United Kingdom.	D
30	Scheuer SH, Fleetwood K, Wild SH, Jackson CA. Ethnic disparities in quality of diabetes care in Scotland: A national cohort study. Diabet Med. 2024;41:e15336. doi:10.1111/dme.15336.	Examines ethnic disparities in overall quality of diabetes care in Scotland rather than diabetic eye screening attendance specifically.	B
31	Brodie J, Misra A, Jones CD, Jenkins C, Bachmann MO. Is diabetic retinopathy screening worthwhile among people first diagnosed with diabetes at older ages? A cohort study of Norfolk diabetic retinopathy screening programme. Diabet Med. 2024;41:e15164. doi:10.1111/dme.15164.	UK screening cohort addresses age at diabetes diagnosis and screening yield rather than minority ethnicity or another prespecified inequality-related attendance focus.	A

No.	Report (Vancouver style)	Study-specific reason for exclusion	Category
32	Scanlon PH, Norridge CFE, Prentis D, Holman N, Rankin P, Valabhji J. Effect of the COVID-19 pandemic on diabetic retinopathy and referral levels in the English National Health Service Diabetic Eye Screening Programme. Diabet Med. 2025;42(5):e15518. doi:10.1111/dme.15518.	Examines COVID-19 effects on retinopathy/referral levels and programme activity rather than eligible attendance factors, barriers, facilitators or interventions.	B
33	Harbers A, Davidson S, Eggleton K. Understanding barriers to diabetes eye screening in a large rural general practice: an audit of patients not reached by screening services. J Prim Health Care. 2022;14(3):273-279. doi:10.1071/HC22062.	Audit of barriers to diabetes eye screening conducted in New Zealand rather than the United Kingdom.	D
34	Al-Busaidi IS, Newcombe L, Jani S, Jani R, O'Grady C, MacCormick IJC. Barriers and enablers of access to diabetes eye care in Auckland, New Zealand: a qualitative study. BMJ Open. 2025;15(1):e087650. doi:10.1136/bmjopen-2024-087650.	Qualitative diabetes eye-care barriers/enablers study conducted in New Zealand rather than the United Kingdom.	D
