# Supplementary material for: Diabetic Eye Screening Attendance Among Minority Ethnic and Other Inequality-Affected Populations in the United Kingdom: A Systematic Review of Associated Factors, Barriers, Facilitators and Interventions
Source: Vision (Basel). 2026 Sep 4;10(4):66. doi: 10.3390/vision10040066 (PMC13600017; doi:10.3390/vision10040066)
Supplement: Supplementary file 1 [file vision-10-00066-s001.zip › v8 Table S1 PRISMA Checklist FINAL.pdf]

Supplementary Table S1 for Diabetic Eye Screening Attendance among Minority Ethnic and Other Inequality-Affected Populations in the United Kingdom: A Systematic Review of Associated Factors, Barriers, Facilitators and Interventions

Supplementary Table S1: Completed PRISMA 2020 Checklist for Systematic Reviews

Topic	No.	Item	Location where item is reported
TITLE			
Title	1	Identify the report as a systematic review.	Title page.
ABSTRACT			
Abstract	2	See the PRISMA 2020 for Abstracts checklist	Abstract.
INTRODUCTION			
Rationale	3	Describe the rationale for the review in the context of existing knowledge.	Section 1 (Introduction), particularly the rationale and evidence-gap paragraphs.
Objectives	4	Provide an explicit statement of the objective(s) or question(s) the review addresses.	Section 1 (Introduction), final paragraph; Section 2.1 (Review Design).
METHODS			
Eligibility criteria	5	Specify the inclusion and exclusion criteria for the review and how studies were grouped for the syntheses.	Sections 2.1-2.2; Tables 1-2; synthesis grouping described in Section 2.7.
Information sources	6	Specify all databases, registers, websites, organisations, reference lists and other sources searched or consulted to identify studies. Specify the date when each source was last searched or consulted.	Section 2.3; Table 3; Table A1. Searches were last rerun on 18 August 2026.
Search strategy	7	Present the full search strategies for all databases, registers and websites, including any filters and limits used.	Section 2.3; complete source-specific strategies, limits and supplementary-search methods in Table A1.
Selection process	8	Specify the methods used to decide whether a study met the inclusion criteria of the review, including how many reviewers screened each record and each report retrieved, whether they worked independently, and if applicable, details of automation tools used in the process.	Section 2.4. Two reviewers independently completed title/abstract and full-text screening; disagreements were resolved by discussion. No automation was used.
Data collection process	9	Specify the methods used to collect data from reports, including how many reviewers collected data from each report, whether they worked independently, any processes for obtaining or confirming data from study investigators, and if applicable, details of automation tools used in the process.	Section 2.5. H.K. extracted the data and F.A. checked them against the source reports; discrepancies were resolved by discussion. No automation was used.

Topic	No.	Item	Location where item is reported
Data items	10a	List and define all outcomes for which data were sought. Specify whether all results that were compatible with each outcome domain in each study were sought (e.g. for all measures, time points, analyses), and if not, the methods used to decide which results to collect.	Sections 2.5 and 2.7. Outcomes included attendance/uptake, disease outcomes, barriers/facilitators, professional perceptions and intervention effects.
	10b	List and define all other variables for which data were sought (e.g. participant and intervention characteristics, funding sources). Describe any assumptions made about any missing or unclear information.	Section 2.5. Other variables included study design, setting, population/sample, equity dimensions, associated factors, intervention details, effect estimates and limitations.
Study risk of bias assessment	11	Specify the methods used to assess risk of bias in the included studies, including details of the tool(s) used, how many reviewers assessed each study and whether they worked independently, and if applicable, details of automation tools used in the process.	Section 2.6; Tables A3-A7. Two reviewers independently applied design-specific Joanna Briggs Institute, Newcastle-Ottawa Scale and Cochrane Risk of Bias 2 tools.
Effect measures	12	Specify for each outcome the effect measure(s) (e.g. risk ratio, mean difference) used in the synthesis or presentation of results.	Section 2.5; Sections 3.4.1-3.4.6 and Table 5 report study-level attendance proportions, odds ratios, percentage-point differences and 95% confidence intervals where available.
Synthesis methods	13a	Describe the processes used to decide which studies were eligible for each synthesis (e.g. tabulating the study intervention characteristics and comparing against the planned groups for each synthesis (item 5)).	Sections 2.2 and 2.7. Evidence was separated by outcome/evidence type and then grouped by recurring equity and service themes; contributing studies are identified in Table 7.
	13b	Describe any methods required to prepare the data for presentation or synthesis, such as handling of missing summary statistics, or data conversions.	Section 2.7. Adjusted estimates were prioritised where available; attendance, disease and perception outcomes were kept distinct. No imputation or substantive data conversion was undertaken.
	13c	Describe any methods used to tabulate or visually display results of individual studies and syntheses.	Figure 1; Tables 4-8; Figure 2; detailed appraisal Tables A3-A7.
	13d	Describe any methods used to synthesize results and provide a rationale for the choice(s). If meta-analysis was performed, describe the model(s), method(s) to identify the presence and extent of statistical heterogeneity, and software package(s) used.	Section 2.7. Structured narrative synthesis was selected because designs, populations, exposures and outcomes were not sufficiently comparable for meta-analysis.

Topic	No.	Item	Location where item is reported
	13e	Describe any methods used to explore possible causes of heterogeneity among study results (e.g. subgroup analysis, meta-regression).	Section 2.7. No formal subgroup analysis or meta-regression was feasible; variation was examined narratively by design, population, setting, exposure and outcome.
	13f	Describe any sensitivity analyses conducted to assess robustness of the synthesized results.	Not applicable: no pooled quantitative synthesis or sensitivity analysis was conducted (Section 2.7).
Reporting bias assessment	14	Describe any methods used to assess risk of bias due to missing results in a synthesis (arising from reporting biases).	No formal reporting-bias assessment was feasible because 14 heterogeneous studies were included and no meta-analysis was performed; the rationale and implications are reported in Section 4.8.
Certainty assessment	15	Describe any methods used to assess certainty (or confidence) in the body of evidence for an outcome.	Section 2.8. Confidence was judged by methodological limitations, directness, consistency, precision and dataset independence; judgements are reported in Table 8.
RESULTS			
Study selection	16a	Describe the results of the search and selection process, from the number of records identified in the search to the number of studies included in the review, ideally using a flow diagram.	Section 3.1; Figure 1; Table 4. Of 1,012 records, 583 were screened, 48 full-text reports were assessed and 14 primary studies were included.
	16b	Cite studies that might appear to meet the inclusion criteria, but which were excluded, and explain why they were excluded.	Section 3.1; Supplementary Table S2 lists all 34 excluded full-text reports with study-specific reasons.
Study characteristics	17	Cite each included study and present its characteristics.	Section 3.2; Table 5. All 14 included primary studies [15-28] are cited and characterised.
Risk of bias in studies	18	Present assessments of risk of bias for each included study.	Section 3.3; Table 6; Figure 2; full study-level assessments in Tables A3-A7.
Results of individual studies	19	For all outcomes, present, for each study: (a) summary statistics for each group (where appropriate) and (b) an effect estimate and its precision (e.g. confidence/credible interval), ideally using structured tables or plots.	Table 5; Sections 3.4.1-3.4.6; Table 7. Group statistics, effect estimates and 95% confidence intervals are reported where available.
Results of syntheses	20a	For each synthesis, briefly summarise the characteristics and risk of bias among contributing studies.	Sections 3.3-3.4; Tables 5-7; Figure 2. Contributing studies and their methodological limitations are identified for each theme.
	20b	Present results of all statistical syntheses conducted. If meta-analysis was done, present for each the summary estimate and its precision (e.g. confidence/credible interval) and measures of statistical heterogeneity. If comparing groups, describe the direction of the effect.	Not applicable: no meta-analysis or pooled statistical synthesis was conducted (Section 2.7). Study-level quantitative estimates are reported in Sections 3.4.1-3.4.6.

Topic	No.	Item	Location where item is reported
	20c	Present results of all investigations of possible causes of heterogeneity among study results.	No formal statistical investigation of heterogeneity was conducted. Variation is examined narratively in Section 3.4 and Sections 4.1-4.8.
	20d	Present results of all sensitivity analyses conducted to assess the robustness of the synthesized results.	Not applicable: no sensitivity analyses were conducted because there was no pooled quantitative synthesis (Section 2.7).
Reporting biases	21	Present assessments of risk of bias due to missing results (arising from reporting biases) for each synthesis assessed.	No formal reporting-bias assessment was conducted; the reason and implications are reported in Section 4.8.
Certainty of evidence	22	Present assessments of certainty (or confidence) in the body of evidence for each outcome assessed.	Table 8; Section 2.8; Section 5. Confidence is reported separately for the principal findings.
DISCUSSION			
Discussion	23a	Provide a general interpretation of the results in the context of other evidence.	Section 4.1 and thematic interpretation in Sections 4.2-4.6; Section 5.
	23b	Discuss any limitations of the evidence included in the review.	Section 4.8. Limitations include heterogeneous designs/outcomes, observational evidence, broad or missing demographic data, overlapping cohorts, small samples, limited intervention evidence and geographical gaps.
	23c	Discuss any limitations of the review processes used.	Section 4.8. Review-process limitations include non-registration, English-language and 2006-onward limits, restricted Google Scholar screening, incomplete grey-literature coverage, and no formal reporting-bias assessment, Grading of Recommendations Assessment, Development and Evaluation assessment, or meta-analysis.
	23d	Discuss implications of the results for practice, policy, and future research.	Section 4.7 (practice and policy); Section 4.9 (future research).
OTHER INFORMATION			
Registration and protocol	24a	Provide registration information for the review, including register name and registration number, or state that the review was not registered.	Sections 2.1 and 4.8. The review was not prospectively registered because registration was overlooked and review activities had already begun; implications and mitigation are stated.
	24b	Indicate where the review protocol can be accessed, or state that a protocol was not prepared.	Not applicable: no separate formal protocol document was prepared. Registration/protocol transparency is addressed in Sections 2.1 and 4.8.
	24c	Describe and explain any amendments to information provided at registration or in the protocol.	Not applicable to a registration record or formal protocol. The broadened search rerun on 18 August 2026 is documented in Sections 2.3 and 4.8.

Topic	No.	Item	Location where item is reported
Support	25	Describe sources of financial or non-financial support for the review, and the role of the funders or sponsors in the review.	Funding statement: no external funding was received.
Competing interests	26	Declare any competing interests of review authors.	Conflicts of Interest statement: the authors declare no conflicts of interest.
Availability of data, code and other materials	27	Report which of the following are publicly available and where they can be found: template data collection forms; data extracted from included studies; data used for all analyses; analytic code; any other materials used in the review.	Data Availability Statement; extracted evidence in Tables 5-8 and Tables A3-A8; study-specific full-text exclusions in Supplementary Table S2. No new participant data or analytic code were generated.

Adapted from: Page MJ, McKenzie JE, Bossuyt PM, Boutron I, Hoffmann TC, Mulrow CD, et al. The PRISMA 2020 statement: an updated guideline for reporting systematic reviews. BMJ. 2021;372:n71. doi:10.1136/bmj.n71. Checklist available at: https://www.prisma-statement.org/
